# The synchrotron radiation source PETRA III and its future ultra-low-emittance upgrade PETRA IV

**DOI:** 10.1140/epjp/s13360-022-03517-6

**Published:** 2022-12-06

**Authors:** Christian G. Schroer, Hans-Christian Wille, Oliver H. Seeck, Kai Bagschik, Horst Schulte-Schrepping, Markus Tischer, Heinz Graafsma, Wiebke Laasch, Karolin Baev, Stephan Klumpp, Riccardo Bartolini, Harald Reichert, Wim Leemans, Edgar Weckert

**Affiliations:** 1 Deutsches Elektronen-Synchrotron DESY, Notkestr. 85, 22607 Hamburg, Germany; 2 Department Physik, Universität Hamburg, Luruper Chaussee 149, 22761 Hamburg, Germany

## Abstract

PETRA III at DESY is one of the brightest synchrotron radiation sources worldwide. It serves a broad international multidisciplinary user community from academia to industry at currently 25 specialised beamlines. With a storage-ring energy of 6 GeV, it provides mainly hard to high-energy X-rays for versatile experiments in a very broad range of scientific fields. It is ideally suited for an upgrade to the ultra-low emittance source PETRA IV, owing to its large circumference of 2304 m. With a targeted storage ring emittance of $20 \times 5\,\textrm{pm}^{2}\,\textrm{rad}^{2},$ PETRA IV will reach spectral brightnesses two to three orders of magnitude higher than today. The unique beam parameters will make PETRA IV the ultimate in situ 3D microscope for biological, chemical, and physical processes helping to address key questions in health, energy, mobility, information technology, and earth and environment.

## Introduction to the facility

**Fig. 1:**
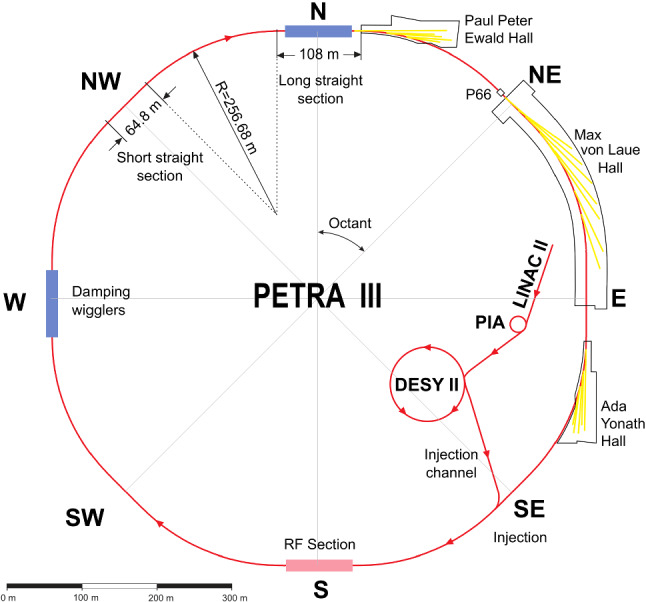
PETRA III: the storage ring with 2304 m circumference hosts 25 beamlines (as of 2021) in three experimental halls from the North to the East of the facility. The pre-accelerator chain is with LINAC II, PIA, and DESY II is also shown

PETRA III at Deutsches Elektronen-Synchrotron DESY in Hamburg, a research centre of the Helmholtz Association in Germany, is one of the brightest storage-ring-based X-ray sources for high-energy photons worldwide. It provides the environment for specialised experiments (cf. Table 1) to address many of the pressing grand challenges of the twenty-first century in energy, life and health, earth and environment, mobility, and information technology. With a circumference of 2304 m it is the largest storage-ring-based source worldwide and operates at an electron energy of 6 GeV. A list of basic storage-ring parameters is shown in Table 2. PETRA III offers experimental techniques that exploit predominantly hard and high-energy X-rays on currently 25 beamlines located in three separate experimental halls (cf. Fig. 1). The beamlines are optimised for various applications employing nano-focused or highly collimated X-ray beams. Three of the beamlines are used and operated by the European Molecular Biology Laboratory (EMBL). Two additional beamlines for materials science are used and operated by the Helmholtz-Zentrum Hereon. The excellent research perspectives at PETRA III are underlined by numerous national and international collaborations, e.g. with the Max Planck Society, the Swedish Research Council, and the Indian Jawaharlal Nehru Center of Advanced Sciences.

The users of PETRA III cover many areas of science and engineering ranging from physics, chemistry, biology and biomedicine, to geological, environmental and material sciences, as well as nanoscience and nanotechnology. In line with the Helmholtz mission, they aim to contribute to solutions of the grand societal challenges, e.g. to finding new drugs or to optimising energy conversion. In 2019, before the Covid-19 pandemic, PETRA III had more than 6300 visits from 3150 national and international users at 21 beamlines operational at that time.

In 2016, DESY proposed the PETRA IV project to upgrade PETRA III to an ultra-low emittance source of the fourth generation. The first part of the project comprises the construction of a new experimental hall (PXW) and office space in the western part of the DESY campus and various support buildings, a new accelerator complex with a storage ring based on a Hybrid Six-Bend Achromat (H6BA) lattice with damping undulators as its core elements. The second part entails the upgrade of existing and the construction of new beamlines for optimum exploitation of the unique beam properties. Due to the large circumference of the storage ring, a crucial prerequisite for achieving brighter beams, PETRA IV will outperform all similar facilities currently planned or in operation, thus putting it in a prime position to drive science and innovation. As the ultimate in situ 3D microscope for biological, chemical and physical processes it will make important contributions to solving the grand societal challenges.

## Current status of PETRA III and its scientific programme

### Status of PETRA III and user operation

PETRA III started user operation in 2010 with 3 beamlines in the ‘Max von Laue Hall’, ramping up the user program at 15 beamlines until the end of 2013 (cf. Fig. 1). The PETRA III extension project, starting in February 2014, added two experimental halls, ‘Ada Yonath’ in the East and ‘Paul Peter Ewald’ in the North (cf. Fig. 1), to accommodate 11 additional insertion-device beamlines (9 as part of the extension project and two free slots for future use). For the civil construction and the upgrade of the storage ring in the concerned octants, PETRA III was shut down for 13 months in March 2014. Since 2015, nine additional insertion-device beamlines and one VUV beamline went into operation. Including this VUV beamline, PETRA III currently offers user service at 25 beamlines with a machine availability in excess of 98%.

Since the PETRA III extension programme was officially completed in 2021, DESY has become increasingly involved in biological and medical research. In this context, the hierarchical imaging experiment (HIKA) at beamline P23 operated by the Karlsruhe Institute of Technology (KIT) in Karlsruhe, Germany, will be commissioned in spring 2023. It is complemented by various X-ray fluorescence imaging methods to be implemented at beamline P25 dedicated to applied biomedical imaging, powder diffraction, and methods and instrumentation developments in view of PETRA IV. This beamline is funded by the Innovation and Technology Transfer (DESY-ITT) department to foster industrial and industry-related research and innovation. It will become operational in the first half of 2024. In addition, P63 is planned as a beamline for catalysis funded by the Max Planck Society.

**Fig. 2:**
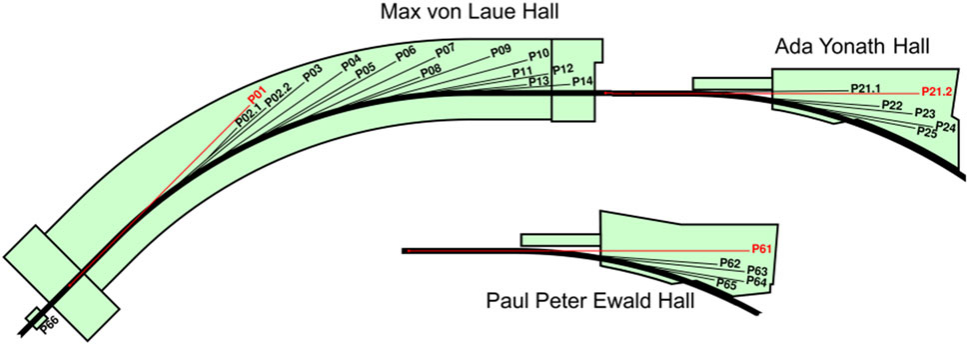
Distribution of PETRA III insertion device (ID) beamlines across the three experimental halls. Beamline sectors marked in red are fed by very long straight sections of the storage ring accommodating very long IDs

Table 1 lists the PETRA III beamlines. Their arrangement in the three experimental halls is shown in Fig. 2. 15 beamlines are distributed over nine sectors in the experimental hall ‘Max von Laue', using a 5 mrad canting in five of the sectors. The two experimental halls ‘Ada Yonath' and ‘Paul Peter Ewald' accommodate six and five beamlines in three sectors, respectively, using a 1 mrad canting at beamlines P21.1 and P21.2 and 20 mrad canting in the other sectors. The relatively short sector length of 23 m compared to other high-energy synchrotron radiation sources and the canting are crucial to accommodate the beamline portfolio of PETRA III in the experimental halls. However, this dense arrangement of the beamlines poses specific challenges for an upgrade to an ultra-low emittance source (cf. Sect. 3).

**Table 1:** PETRA III beamline portfolio

Beamline	Techniques	Energy range
**High-Energy X-rays**		
P02.1 powder diffraction and total scattering	XRD (powder), PDF, TS	60 keV
P02.2 extreme conditions	XRD (SC, powder, amorphous)	25.6, 42.6 keV
P07 high-energy material science (Hereon)	XRD, PDF, SAXS, 3DXRD, Tomogr.	50–200 keV
P21.1 Swedish materials science beamline	XRD	50, 80, 100 keV
P21.2 Swedish materials science beamline	XRD, SAXS, WAXS, Imaging	40–150 keV
P61 high-energy wiggler beamline LVP	XRD, Radiography	30–250 keV
**X-ray scattering and diffraction**		
P03 MiNaXS	(U)SAXS, (GI)WAXS, GISAXS	7–21 keV
P08 high resolution diffraction	XRD, GID, SAXS, XRR	5.4–29.4 keV
P23 In situ X-ray diffraction and imaging	XRD, XANES, (GI)SAXS	5–35 keV
P62 SAXSMAT	(A)SAXS, GISAXS, (A)WAXS	3.5–35 keV
P24 chemical crystallography	XRD (single crystal)	8, 15–44 keV
**X-ray imaging and coherence**		
P10 coherence applications	XPCS, XCCA, BCDI, Holotomo.	4–20 keV
P05 imaging beamline (Hereon)	FF-tomography, $\mu$- and nano-Tomo.	8–50 keV
P06 Hard X-ray micro-/nanoprobe	$\mu$-XRF, $\mu$-XRD, $\mu$-XAS	5–45 keV
P25$^1$ Appl. Bio-Med. Img., Powder Diffr. & Innov.	XRF, XRD (powder)	8–60 keV
**X-ray spectroscopy**		
P01 high resolution dynamics (MPG)	NRS, IXS, RIXS	2.5–80 keV
P64 advanced XAFS	EXAFS, QEXAFS, RXES	4–44 keV
P65 applied XAFS	EXAFS, XANES	4–44 keV
P09 resonant scattering and diffraction	REXS, XRMR, XMCD	2.7–31 keV
P22 Hard X-ray photoelectron spectroscopy	HAXPES, HAXPEEM, k-Micros.	2.4–15 keV
P63$^2$ OperandoCat (MPG)	SAXS, XRD Q-EXAFS	not specified
**Structural Biology**		
P11 high-throughput macromolecular crystallogr.	MX	2.4–30 keV
P12 BioSAXS (EMBL)	SAXS	4–20 keV
P13 macromolecular crystallogr. (EMBL)	MX	4.5–17.5 keV
P14 macromolecular crystallogr. and Img. (EMBL)	MX	6–20 keV
**VUV and Soft X-rays**		
P04 variable polarisation XUV beamline	ARPES, RIXS, STXM	0.25–2.8 keV
P66 Superlumi	UV - VUV spectroscopy	0.004–0.04 keV

Partner institutions for specific beamlines are given in parenthesis. The beamlines are sorted into six categories that are also used to categorise the future PETRA IV beamlines. $^1$Under construction, $^2$in planning

PETRA III serves users from both science and industry, providing access within the general user programme, special collaborations, and privileged access. Within the general user programme, new evaluation criteria were introduced, addressing potential socio-economic impact of the proposed research. The user community is very diverse, and PETRA III caters to all major scientific organisations, i. e., the Helmholtz Association, Max Planck Society, Leibnitz Association, (inter-)national universities, and many other institutions as well as industry. Beamtime at PETRA III is in high demand with an average overbooking by a factor of two to three. Over the last two years, PETRA III has operated under special pandemic conditions, increasing the number of remote access and mail-in experiments wherever possible.

To enable and strengthen a science programme targeted towards solving major societal challenges, DESY has introduced new access modes. The first new mode is a ‘Targeted Challenge-Driven Access’ and is open to consortia of at least three independent research groups, collaborating on a topic of scientific relevance. The first call in 2022 focusing on ‘Molecular Water Science’ was answered by several consortia. The call addresses water as a key element to life on our planet, playing a central role in a large number of environmental and technological processes starting from the complex aqueous contents of cells, to clouds in the atmosphere and aqueous catalytic cycles in industry. Following peer review, the first joint long-term projects (with a duration of two years) will start in the first half of 2023.

The second new access mode focuses on ‘Immediate Socio-Economic Impact’ and is open to users working on topics of societal or applied relevance. Granting rapid access to beamtime is an additional feature of this mode of particular importance in applied or industrial research. Here, proposals are evaluated not in terms of the scientific merit but of technological readiness level and impact to society.

### PETRA III scientific programme

PETRA III serves a broad user community, covering a very broad range of scientific fields, from physics [1–4], chemistry [5, 6], and biology, to medicine [7, 8], materials [9–11], geo- [12–15] and environmental [16, 17] science, archaeometry [18], and nanotechnology [19, 20]. With this, it makes an important contribution to solving the grand societal challenges, e.g. in human health [21, 22], energy [23, 24], mobility, information, and earth and environment and ranges from fundamental science [1–3, 25, 26] to industrial applications [9, 27–30]. A key strength is to perform experiments in situ and under working conditions, giving insight into processes, e.g. in catalysis [5, 6] or materials synthesis [10, 31, 32]. To date, more than 4700 publications (average journal impact factor 6.0) resulted from research at PETRA III, 23% of which published in journals with an impact factor larger than 7. The publication output has been steadily increasing, reaching more than 890 publications in 2021.

During the Covid-19 pandemic, PETRA III was fully operational for external users at all times, following strict hygiene rules. In 2020 and 2021, PETRA III has, for instance, contributed to challenge-driven research, by (i) searching for anti-viral drugs by massive drug screening [22] and for synthetic nanobodies against SARS-CoV-2 [33], (ii) supporting vaccine development by studies concerning drug delivery [28–30] at EMBL beamline P12 in collaboration with the German biopharmaceutical company BioNTech, and by (iii) giving insight into the histology of lung [34] and heart tissue, recovered from Covid-19 victims, by tomographic phase-contrast microscopy [21].

## Technological developments and upgrade plans

### Strategic need for sources with high brightness

The solutions to many societal and economic challenges such as the ones cited in Sect. 2.2 (e.g. technologies for circular economy or next generation information systems) will crucially depend on the availability of novel materials with tailor-made functions. Realising these requires sophisticated materials’ design ultimately down to the level of individual atoms, which in turn can only be accomplished if suitable diagnostics tools are available. Similarly, a deep understanding of complex physiological processes is needed to, e.g. enable targeted drug delivery in the overall quest to improve health care.

Revolutionary new analytical capabilities are essential for observing mechanisms down to the atomic scale in natural and technological processes with highest precision, and to achieve a fundamental understanding that allows the transition from an often empirical trial-and-error approach of synthesis to knowledge-based rational design of materials and pharmaceuticals or processes. This requires a new generation of high-energy photon sources with transformative analytical capabilities.

The multibend achromat (MBA) lattice concept enables such a new generation of X-ray sources. The synchrotron radiation source MAX IV in Lund (Sweden) was the first light source to be successfully commissioned with this new lattice type. In 2020, the ESRF-EBS in Grenoble (France) has taken up user operation as the first high-energy ultra-low emittance source employing the hybrid-multibend achromat (HMBA) concept as the next step in storage-ring technology. In general, the (H)MBA lattice provides an increase in spectral brightness by one to two orders of magnitude, which disruptively changes the landscape of synchrotron radiation facilities in the next decade. The operators of all the major existing synchrotron radiation sources worldwide are developing or already implementing plans to upgrade to this new technology. Several new facilities are planned or under construction.

### The PETRA IV project

It is the aim of the PETRA IV project to make best use of all the latest technologies available, such as this new lattice type. This will result in a transformative increase in brightness by two to three orders of magnitude, approaching the physical diffraction limit for hard X-rays up to 10 keV. The high brightness and high degree of spatial coherence allow for efficient focusing of the X-rays to the nanoscale, giving access to local molecular and electronic structures and processes in vivo, in situ and operando, non-destructively and with highest resolution and sensitivity. This effectively makes PETRA IV the ultimate 3D X-ray microscope to study biological, chemical, and physical processes. The project is part of the ‘Helmholtz Photon Science Roadmap’ [35] that describes the strategic upgrade plans for large-scale photon sources in Germany operating as user facilities.

**Fig. 3:**
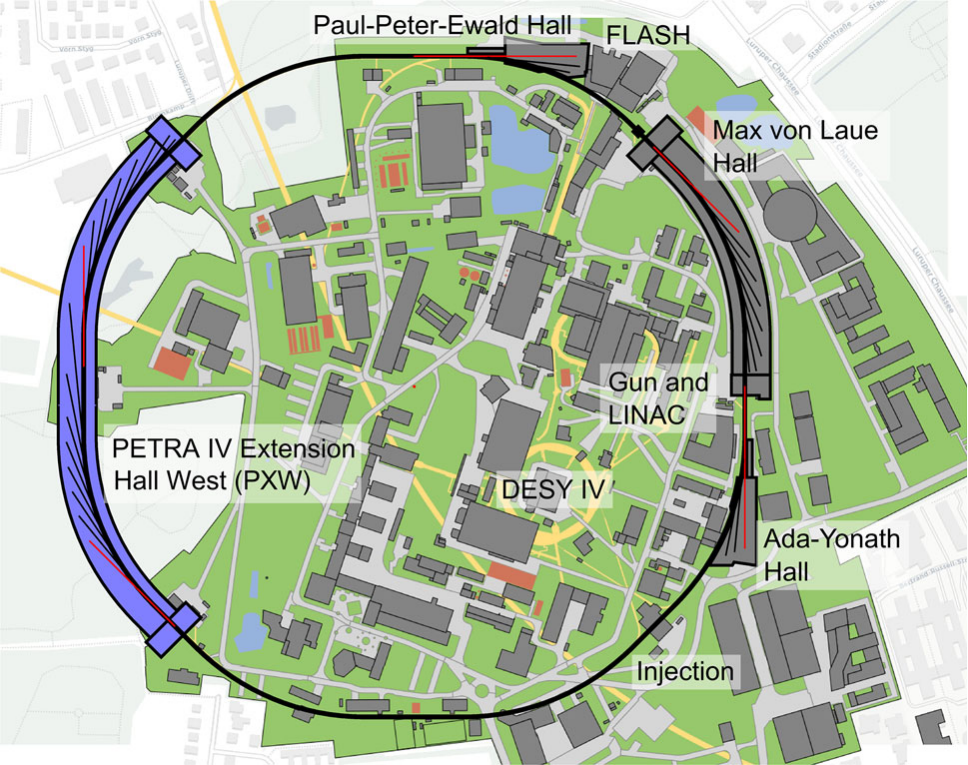
PETRA IV accelerator complex and the four experimental halls, including the planned new PETRA IV Extension Hall West

Due to the fact that the horizontal emittance scales favourably with storage ring size and PETRA’s particularly large circumference of 2304 m, the PETRA complex offers the unique opportunity to push the generation of synchrotron radiation to its physical limits. With a targeted horizontal emittance of 20 pm rad and a vertical emittance of 5 pm rad, PETRA IV would reach the diffraction limit for X-rays up to 10 keV. Figure 3 shows the layout of the planned synchrotron radiation facility PETRA IV.

The planning of PETRA IV started in 2016 with the conceptual design phase [36] that ended with the publication of the Conceptual Design Report (CDR) [37] in 2019. It gives a detailed account of the science case and describes a conceptual design of the storage ring based on a hybrid seven-bend achromat (H7BA) lattice. Since 2020, the project is in the technical design phase, developing detailed designs for the new facility that will be published in the Technical Design Report (TDR) in 2023. In parallel, DESY is preparing the funding proposal to be presented to the German Federal Ministry of Education and Research (BMBF). The current schedule for the project calls for a preparatory phase from 2023 to the end of 2026, during which the procurement of components and preparation of logistics will take place. With the planned shutdown of PETRA III at the end of 2026, the two year long construction phase will start. During that time, the experimental hall PXW in the western part of the DESY campus will be constructed (cf. Fig. 3). The pre-accelerator chain will be refurbished and the current booster DESY II will be replaced by the (low-emittance) booster synchrotron DESY IV. The PETRA III storage ring will be dismantled and replaced by the new storage ring, including all front-ends. In parallel, the beamlines in the existing experimental halls will be adapted to the new source (cf. Sect. 3.4). Current planning foresees the start of PETRA IV operation in early 2029. Concomitantly, the construction phase for the beamlines in the new experimental hall PXW will start and last until 2033. Furthermore, the possibility of adding a new injector based on a laser plasma accelerator operating at 6 GeV is already foreseen with dedicated R&D for this emerging technology underway [38, 39].

### PETRA IV storage ring

The conceptual design of the PETRA IV storage ring was initially based on a H7BA lattice as mentioned in the last section. In 2021, a modified lattice type was introduced. The previous baseline design, the so-called H7BA combi lattice, was replaced by a Hybrid Six-Bend Achromat (H6BA) lattice with additional damping undulators. This strategic decision was triggered by the superior performance of the new lattice in terms of machine parameters and beam stability (see Table 2) as well as its compatibility with the current arrangement of beamlines on the PETRA III experimental floor. The larger natural emittance of the H6BA lattice (45 pm rad) is reduced to the target emittance of 20 pm rad by using damping undulators. Their use has the additional advantage of providing efficient and reliable emittance control. The bare H6BA lattice features a remarkably larger dynamic aperture and momentum acceptance compared to the previous baseline lattice. The result is a storage ring with a much larger Touschek lifetime and stability, enabling operation with off-axis injection and accumulation, which could not be guaranteed for the previous lattice design. The target parameters for the H6BA lattice of PETRA IV are summarised in Table 2 and compared to the current parameters of PETRA III.

**Table 2:** PETRA IV parameters compared to PETRA III

Parameter	PETRA IV		PETRA III	
Operation Mode	Brightness	Timing	Continuous	Timing
Storage ring energy	6 GeV		6 GeV	
Storage ring circumference	2304 m		2304 m	
Number of bunches	1600–1920	80 (40)	480–960	40
Total current / mA	200	80 (80)	120	100
Bunch current / mA	0.125	1.0 (2.0)	0.25–0.125	2.5
Arc ID $\beta _x$/$\beta _y$ / m	2.2/2.2		high $\beta$: 20.0 / 4.0, low $\beta$: 1.4 / 4.0	
Long ID $\beta _x$/$\beta _y$ / m	4.0/4.0		high $\beta$: 16 / 5.0	
Emittance				
Horiz. $\epsilon _{x}$ / pmrad	20	35 (38)	1300	
Vert. $\epsilon _{y}$ / pmrad	5	7 (8)	10	
Bunch length $\sigma _z$ / ps	30	65 (75)	40	43
Bunch separation / ns	4	96 (192)	16–8	192
Energy spread $\sigma _p$ / $10^{-3}$	0.9	1.2 (1.5)	1.3	1.3
Beam lifetime $\tau$ / h	$>10$	$>5$	9–13	1.5
Number of beamlines	35 + 1 VUV		24 (26) + 1 VUV	

PETRA IV will feature two modes of operation, one optimised for highest brightness and one enabling time-resolved experiments

**Fig. 4:**
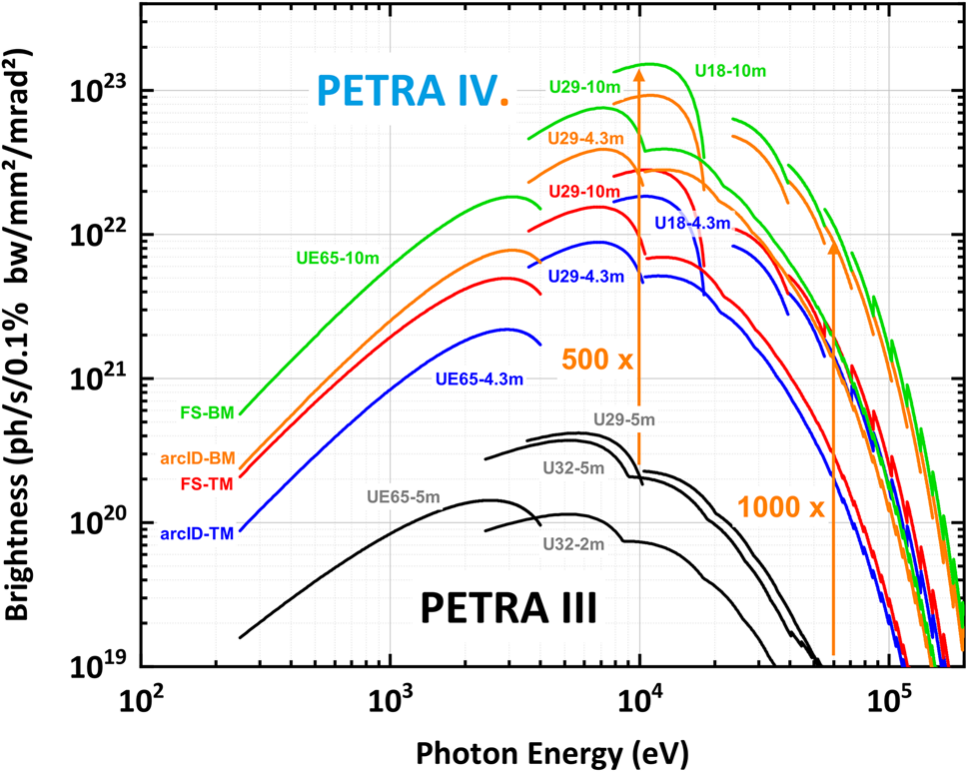
Spectral brightness of PETRA IV (H6BA lattice) compared to PETRA III. The colours denote different modes of operation and ID-cell lengths. The green, orange, red, and blue colours correspond to a 10 m insertion device (ID) in brightness mode, a 4.3 m ID in brightness mode, a 10 m ID in timing mode, and a 4.3 m ID in timing mode, respectively

The new design has five octants (arcs) with nine 22.75 m long H6BA cells with straight sections for damping undulators where no beamlines are located and insertion devices (IDs) for beamlines in the two existing extension halls. The remaining three octants each consist of nine 23 m long H6BA cells with straight sections for IDs in the ‘Max von Laue’ hall and the new PXW hall (Fig. 3). The geometry of the sectors in the ‘Max von Laue’ hall remains unchanged, allowing to reuse large parts of the existing infrastructure. Similarly, the geometries of five beamlines in the two existing extension halls can be nearly preserved. In order to reach the low emittance, the canting of beamlines will be limited to a minimum. 5 mrad canting is planned for the two structural biology sectors at the end of the ‘Max von Laue’ hall (today: P11–P14) and a 1 mrad canting for the Swedish Materials Science sector (today: P21) in the ‘Ada Yonath’ hall. Thus, 33 straight sections will be available for 36 beamlines. 28 straight sections can accommodate IDs with a length of 4.3 m, while five straight sections provide space for 10 m IDs. With optimised insertion devices, PETRA IV will exceed the PETRA III spectral brightness by about a factor 500 at 10 keV and by a factor 1000 at 60 keV. Figure 4 shows the brightness achievable at PETRA IV.

### PETRA IV beamlines

The scientific case including the unique beam properties of PETRA IV as well as the needs of the user community at large were the starting point for the development of a beamline portfolio. Starting in early 2020, the international synchrotron radiation user community and interested scientists were invited to submit proposals for scientific instruments that would advance their science while making best use of PETRA IV. The development of these scientific instrumentation proposals (SIPs) was accompanied by a series of workshops to foster collaboration and promote experimental techniques across fields. In the following step, the SIPs were peer reviewed, grouped and integrated into beamlines by experts from PETRA III and DESY partners. The resulting beamline portfolio was refined together with DESY’s Photon Science Committee (PSC) and internationally reviewed with a focus on the coverage of the PETRA IV science case, user demands, productivity, as well as a well-balanced portfolio of techniques and the possibility for future additions and upgrades. For a comprehensive assessment of the beamline portfolio in terms of coverage of industry-relevant technology trends in the next 10–15 years, an extensive screening of tech-trend and foresight studies was conducted [40]. The result confirmed that the beamline portfolio covers the needs of applied research and industry particularly well. The beamline portfolio encompasses the 28 beamlines listed in Table 3. It serves as the basis for the technical design and the funding proposal, and will be updated and refined continuously throughout the detailed design and construction phase. Considering the dynamic development of economy, society and our environment, the remaining seven free beamline slots provide sufficient flexibility to react to new demands.

**Table 3:** PETRA IV beamline portfolio

Beamline	Techniques	Energy range
**High-energy X-rays**		
Powder diffraction and total scattering	PXRD, TS	15–80 keV
Swedish high-energy material science beamline	WAXS/3DXRD, SAXS, imaging	38–150 keV
High-energy scatt. and diff. tomography	GI-/XRD/-CT, SAXS, TS, CDI	40–120 keV
High-energy material science (Hereon)	XRD/-CT, SAXS	30–200 keV
ExTReM (extreme conditions research)	XRD, PDF, PCI, CDI	25–58 keV
In situ large volume press	AD-/ED-XRD, PXRD, A/PCI	40–130 keV
**X-ray scattering and diffraction**		
AdMiNaXS	GI/T/SAXS/WAXS, CoGISAXS	7–30 keV
SAXSMAT II	(Anom./U)SAXS/WAXS, SAXS Tomo.	5–60 keV
Surface and interface dynamics	GI-XRD, GI-SAXS, XRR	8–40 keV
Chemical crystallography	PXRD, crystallography	15–50 keV
**X-ray imaging and coherence**		
Coherent applications	XPCS, XCCA, Holotomo.	7–25 keV
Materials scanning nanoscope	XRF, XRD, XBIC, XEOL, Ptycho.	2.4–50 keV
In-situ/high-resolution 3D nanoprobe	XRF, XRD, XBIC, XANES, Ptycho.	4–100 keV
CryoBio nanoprobe	Compton Micro., Holotomo., XRF	17–60 keV
In situ bragg microscopy	(GI-)XRD, FFDXM, BCDI	7–40 keV
Full-field imaging for material science (Hereon)	Tomography, radiography	10–200 keV
**X-ray spectroscopy**		
X-ray absorption & emission Spec.	HR-XES/XAS, TR-XES/XAS	4–25 keV
Materials science lab beamline (MPG)	XAFS, XRD/PDF, tomography	2–100 keV
Applied analytical XAFS and Q-EXAFS	XAS, EXAFS, XANES, Q-XAFS	4–45 keV
Nuclear resonance and X-ray Raman scattering	NFS, NIS, SMS, XRS, (R)XES	6.5–73 keV
Resonant X-ray scattering (MPG)	RIXS, REXS	2.4–14 keV
Hard X-ray photoelectron spectromicroscopy	HAXPES(ARPES, PEEM, XPD), CDI	2.4–15 keV
**Structural biology**		
High-throughput and polychromatic MX	MX, SSX	6–30 keV
BioSAXS (EMBL)	BioSAXS, TR-SAXS, HT-SAXS	6–20 keV
High performance and microfocus MX (EMBL)	HT-MX	5–30 keV
Bio diffraction and imaging (EMBL)	MX, TR-MX, HiTT	6–30 keV
**VUV and soft X-rays**		
High-resolution high-stability soft X-ray beamline	ARPES, CDI, STXM-XRF, REMI	0.25–4 keV
Time-resolved VUV spectroscopy	IR-Vis, VUV spectroscopy	4–40 eV

Partner institutions for specific beamlines are given in parenthesis. The beamlines are sorted into six categories (cf. Table 1)

### Beamline technologies

The PETRA IV project is accompanied by several technological developments at DESY, which range from undulator developments, X-ray optics, ultra-precision mechanical systems for beamline components to high-resolution detectors. In addition, many methodological developments are already pursued at PETRA III in view of optimal use of PETRA IV, in particular in the fields of spectroscopy [41], X-ray microscopy [42–47], X-ray coherence applications [48], and data science.

The new H6BA lattice of PETRA IV provides space for up to 4.3 m long undulators. The magnet design for the conventional planar and helical in-air devices will be optimised for the spectral requirements of each beamline and the PETRA IV electron beam parameters via a dedicated programme for the refurbishment of PETRA III undulators. For specific beamlines, new cryogenic permanent magnet undulators (CPMUs) will be developed as well as conventional in-vacuum undulators (IVUs). Experiments requiring high-energy photons will particularly benefit from these new devices.

In the field of X-ray optics, refractive lenses (CRLs) and multilayer Laue lenses (MLLs) [49] are intensively developed at DESY. MMLs in particular can generate single-digit nanometre foci [50] and reach, at least in principle, a focal spot size of one nanometre [51, 52]. DESY is also developing schemes to tailor X-ray optical wavefronts, be it for aberration correction of X-ray optical systems [53–55] or the creation of unusual X-ray wave fields such as beams with orbital angular momentum [56]. These optics and optical schemes are important technologies for X-ray microscopy at PETRA IV.

Two new detector systems are under development specifically for PETRA IV. The first system is based on the new TimePix4 chip [57] and offers two different operation modes. The first one is a classical frame-based readout, with a frame rate of 40 kHz, with a possible extension to 80 kHz, and pixel count-rates up to $15\times 10^6$ counts per second. Alternatively, the system can be operated in an event-driven mode, where every incoming photon is precisely time-stamped with nanosecond resolution. The second system under development is CoRDIA [58]. This system uses the AGIPD technology, with adaptive gains to provide both low noise and large dynamic range. It will feature a pixel size of 100 µm and provides continuous frame-rates exceeding 130 kHz.

## Perspectives for science and innovation

The world is currently facing major global challenges in the fields of health, energy, environment, mobility and information technology. Examples include the fight against cancer and infectious diseases, the supply of clean and sustainable energy as well as the reduction of greenhouse gases. From a scientific point of view, solutions to these problems are closely related to our understanding of the structures and processes in matter on different length scales from atomic distances to macroscopic dimensions and of the underlying fundamental interactions. A current lack of knowledge about the connection between the microscopic properties of matter on one side and the macroscopic behaviour of materials on the other side still prevents significant progress and targeted improvement in these areas. The scale-bridging mapping of structures and their dynamics under in vivo and in situ/operando conditions is therefore essential to decipher the processes and functionalities that occur in nature and technology and thus contribute to finding solutions for important aspects of our grand challenges.

This can be particularly well achieved with the highly-brilliant X-rays of PETRA IV, whose large spatial coherence length will enable high-resolution 3D imaging of materials in real time and under working conditions. The combination of PETRA IV instrumentation with complementary analytical methods provided by other large-scale facilities and infrastructures available at DESY and on the campus in Hamburg, such as FLASH (including its upgrade programme FLASH 2020+), Cryo Electron Microscopes (Cryo EMs), and the DESY NanoLab, offers a unique scale-bridging approach to solving critical questions in the natural and engineering sciences. Making use of both the European XFEL and PETRA IV, scientists will be able to study the dynamics in condensed matter from femtoseconds to hours on all relevant length scales.

A high degree of standardisation and automation enables very high throughput at many beamlines and instruments. This is particularly relevant for industrial users. To support industrial users and applied research at PETRA IV, an innovation ecosystem is currently being established at the DESY site in context of the Science City Hamburg Bahrenfeld planned in the area. These infrastructures provide support and incubators for start-ups and spin-offs, room for innovative collaboration projects with industry and space for high-tech companies that need a close contact to scientific infrastructures, in particular PETRA IV. A very important and central aspect is direct and flexible access to PETRA IV on short notice for commercial users, minimising administrative steps and providing service options such as mail-in services and remote access to the beamlines. In this context, DESY is currently developing a modern service concept on selected beamlines at PETRA III to better support academic and commercial users with ancillary infrastructures on the experimental floor for the preparation of experiments, data collection, and data analysis/interpretation. This support will be fully rolled out at PETRA IV.

## Data Availability

No Data associated in the manuscript.
